# *GM *and *KM *immunoglobulin allotypes in the Galician population: new insights into the peopling of the Iberian Peninsula

**DOI:** 10.1186/1471-2156-8-37

**Published:** 2007-06-27

**Authors:** Rosario Calderón, Rosa Lodeiro, Tito A Varela, José Fariña, Beatriz Ambrosio, Evelyne Guitard, Antonio González-Martín, Jean M Dugoujon

**Affiliations:** 1 Departamento de Zoología y Antropología Física, Facultad de Biología, Universidad Complutense, Madrid, Spain; 2 Departamento de Biología Animal, Unidad de Antropología Física, Facultad de Biología, Universidad de Santiago de Compostela, Santiago de Compostela, Spain; 3 Departamento de Ecología y Biología Animal, Área de Antropología Física, Facultad de Biología, Universidad de Vigo, Vigo, Spain; 4 Centre d'Anthropologie, FRE 2960, CNRS, Université Paul Sabatier, Toulouse, France

## Abstract

**Background:**

The current genetic structure of Iberian populations has presumably been affected by the complex orography of its territory, the different people and civilizations that settled there, its ancient and complex history, the diverse and persistent sociocultural patterns in its different regions, and also by the effects of the Iberian Peninsula representing a refugium area after the last glacial maximum. This paper presents the first data on *GM *and *KM *immunoglobulin allotypes in the Galician population and, thus, provides further insights into the extent of genetic diversity in populations settled in the geographic extremes of the Cantabrian region of northern Spain. Furthermore, the genetic relationships of Galicians with other European populations have been investigated.

**Results:**

Galician population shows a genetic profile for *GM *haplotypes that is defined by the high presence of the European Mediterranean GM*3 23 5* haplotype, and the relatively high incidence of the African marker GM*1,17 23' 5*. Data based on comparisons between Galician and other Spanish populations (mainly from the north of the peninsula) reveal a poor correlation between geographic and genetic distances (*r *= 0.30, *P *= 0.105), a noticeable but variable genetic distances between Galician and Basque subpopulations, and a rather close genetic affinity between Galicia and Valencia, populations which are geographically separated by a long distance and have quite dissimilar cultures and histories. Interestingly, Galicia occupies a central position in the European genetic map, despite being geographically placed at one extreme of the European continent, while displaying a close genetic proximity to Portugal, a finding that is consistent with their shared histories over centuries.

**Conclusion:**

These findings suggest that the population of Galicia is the result of a relatively balanced mixture of European populations or of the ancestral populations that gave rise to them. This would support the importance of the migratory movements that have taken place in Europe over the course of recent human history and their effects on the European genetic landscape.

## Background

**Figure 1:**
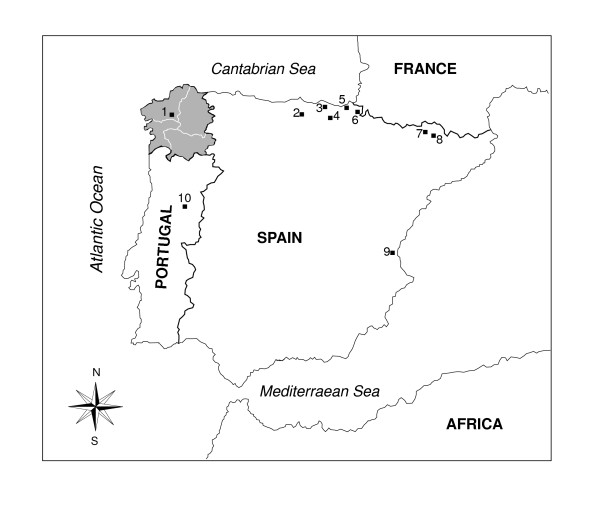
Map of Iberian Peninsula showing the location of the study Galician region (grey) and the locations of sampling sites in mainland Spain, where population data for *GM *and *KM *immunoglobulin allotypes are available. The site numbers correspond to those of Table 2.

Galicia is located at the north-west tip of the Iberian Peninsula and covers an area of 29 424 km^2^, 5.8% of the total area of Spain. It forms part of the Cantabrian mountain range, which extends along the coast of the Cantabrian Sea from Cape Finisterre to the western limit of the Pyrenees. The territory is bordered to the north and west by the Atlantic Ocean, to the south by Portugal and to the east by the regions of Asturias and Leon (Figure 1).

Galicia is currently divided into four provinces: La Coruña, Lugo, Orense and Pontevedra. Its economy has traditionally been based on agriculture and fishing. The population of Galicia has a high level of ethnicity as a result of its extreme geographic position and difficulties of communication with adjacent regions. A distinctive characteristic of the region is that it possesses its own language, *Gallego*, which began to be spoken as a language that could be distinguished from Latin in the 9th Century. Gallego and Portuguese became differentiated from one another as romance languages around the middle of the 14th century.

The presence of megalithic dolmens known as *mámoas *throughout the region provides evidence supporting the prehistoric peopling of Galicia [1]. The existence of substantial deposits of gold, tin, and copper made way for the introduction of metalwork in this megalithic culture, a process that marked the beginning of the Bronze Age in the region, when permanent settlements appeared and commercial relations began via the Atlantic with the British Isles and various Mediterranean populations. Ironwork was introduced to Galicia by the Celts. This people, originating in central Europe, appears to have arrived in the Iberian Peninsula in two phases, the first in the 9th century BC and the second, when they presumably became established in Galicia, around the 6th century BC [2]. The Celts developed the so-called "northern culture of fortified towns or *Castros*" that prevailed in Galicia until the period of Roman domination and, may have been the basis for the current distribution of the population of Galicia [3]. Only some of the pre-Roman languages of the peninsula belonged to the Indo-European group, which extended throughout most of the western and central parts of the peninsula and to which the language spoken at that time in Galicia belonged.

Romanization was an important process in the history of Europe, particularly that of the Iberian Peninsula. The populations in the north of the peninsula avoided Roman domination until the campaign of Augustus in the 1st century BC and maintained some aspects of their indigenous cultures. Roman domination of *Gallaetia *gave rise to important communications routes (e.g. *via XXXIV *in the Itinerary of Antoninus), as well as a new political organization and restructuring of the population through two important cities, Braga (Portugal) and Lugo (Galicia). It should be noted that the main route of communication in the north of the Peninsula and in an east-west direction occurred through the flat areas of the central plateau and not through the mountainous costal areas, which were accessed by branches at various points leading off the main route. Thus, this communication system maintained a certain degree of isolation between nearby coastal populations.

At the beginning of 5th century, when the Roman Empire fell, the Swabians, a people of German origin, settled for a period of time in Galicia. They lived alongside the native population and at the end of 6th century the Swabian kingdom was incorporated into the Visigothic *Hispania*. Both Swabians and Visigoths maintained the sociocultural organization of the Roman Empire.

As occurred with the process of Romanization, the impact of the Muslim invasion of the Iberian Peninsula in the year 711 was less in both Galicia and other northern territories than in other regions. As a consequence, the regions of Galicia, northern Portugal, and the area to the north of the River Duero acted as important refuges from the Muslim invasion during the 9th and 10th centuries. This led to a process of repopulation that was most rapid in the western part of Galicia and Portugal.

Following the Christian reconquest, the kingdom of Asturias and Leon appeared, including part of Galicia. This kingdom was later integrated as part of the Crown of Castile. Following a series of changes, Portugal was recognized as an independent kingdom in 1143. The process of the Christian reconquest also led to significant migration from the north to the south of the peninsula to repopulate the conquered territories, while east-west movement was limited by the existence of different Christian kingdoms. The border between Castile and Portugal caused a significant reduction in the previously unrestricted movement between the old *Roman Galicias *on either side of the border.

Pilgrimages to Santiago de Compostela, one of the main Christian destinations alongside Jerusalem and Rome, have allowed contact between Galicia and numerous peoples of Europe who have continued to arrive since the Middle Ages, largely following the old *vias *of the Antoninus Itinerary, known since then as the Camino de Santiago.

Geography, relief, and climate, as well as history, would have contributed to endowing a certain similarity between the populations of the north of Spain and to distinguishing them from other populations of the peninsula. This observation is supported by analysis of inbreeding patterns. The high rates of marriages between close relatives (uncle-niece/aunt-nephew: M12 and first cousins: M22) and the levels of inbreeding distinguish northern Iberians, namely Galicians, Asturians, Cantabrians and Basques [4-7] from other Spanish populations [8-10].

Current data on the genetic variability of the populations from the Iberian Peninsula are highly interesting and the Cantabrian cornice represents a major component of the anthropogenetic data that has been gathered to date. Genetic variation in the Galician population has been analyzed in relation to most classical polymorphisms (blood groups, enzymes and serum proteins) and, recently, a large number of surveys have been undertaken to assess DNA variation in the Galician genome, based on the Y-chromosome [11-14], *Alu *insertions [15] and mitochondrial DNA (mtDNA) [16], among others. These genetic studies have mainly focused on the extent of genetic affinity between Galicia and other adjacent territories (e.g. Portugal) or outlier populations such as the Basques and to evaluate the impact on the peopling process of the Peninsula through the western Pyrenees, one of the main corridors for the entry of humans to the Iberian Peninsula since prehistoric times.

GM antigenic determinants are present on the heavy chains of 3 of the 4 subclasses of IgG (IgG1, IgG2 and IgG3) [17]. *GM *allotypes are encoded on chromosome 14 (14q32.3) by closely linked alleles which are inherited in fixed combinations called haplotypes. The kappa gene, assigned to chromosome 2 (2p12), encodes for the *KM *system on constant domains of kappa light chains. Data on *GM *and *KM *allotypes of human immunoglobulins in Basques and other groups in the area surrounding the Basque region have been reported previously [18-20]. However, while other facets of the genetic structure of Iberian have been analyzed, analysis of these immunoglobulin markers has not been performed in the Galician population. This paper presents the first data on *GM *and *KM *allotypes in the Galician autochthonous population and thus provides pioneering results on the extent of genetic diversity of populations settled in the geographic extremes of the Cantabrian region of northern Spain.

## Results

Table 1 shows the phenotype and allotype frequencies for the *GM *and *KM *systems in the Galician population. The observed GM phenotype frequencies fit a Hardy-Weinberg equilibrium (χ^2 ^= 6.85, d.f. = 9, n.s.). Fourteen GM phenotypes were detected. The most common were GM 3 23 5* (41.5%) and GM 1,3,17 23 5*,21,28 (20.2%), followed by GM 1,2,3,17 23 5*,21,28 (8.4%), GM 1,3,17 23' 5*,21,28 (7.6%), and GM 1,3,17 23' 5* (5.3%). Other GM phenotypes were only found at very low frequencies. As expected for a European population, the most common KM phenotype was KM (-1) (79.6%) and its corresponding KM*3 allele reached a frequency of 89.2%, which is within the range of European values.

**Table 1:** GM and KM phenotype and allotype frequencies in the Galician population.

	*Absolute*	*Expected*		*Frequency*	*S.E*.
**GM Phenotypes**			***GM Haplotypes***		
3 23 5*	148	153.01	*1,17 23' 21,28*	0.1995	0.0151
3 23' 5*	15	11.71	*1,2,17 23' 21,28*	0.0751	0.0100
1,17 23' 21,28	16	14.20	3 23' 5*	0.1811	0.0181
1,2,17 23' 21,28	10	12.70	3 23 5*	0.4982	0.0217
1,3,17 23 5*,21,28	72	70.95	1,17 23' 5*	0.0448	0.0078
1,3,17 23' 5*,21,28	27	25.79	*1,17 23' 10,11,13,15,16*	0.0014	0.0014
1,2,3,17 23 5*,21,28	30	26.69			
1,2,3,17 23' 5*,21,28	7	9.70	Gene Diversity (h)	0.6729	0.0184
1,3,17 23 5*	19	15.94			
1,3,17 23' 5*	3	5.80			
1,17 23' 5*;21,28	3	6.38			
1,3,17 23 5*,15,16	1	0.50			
1,2,17 23' 5*;21,28	5	2.40			
1,17 23' 5*	1	0.72			
Other phenotype combinations	0	0.50			
					
Sample size	357				
					
	χ^2 ^= 6.85 *d*.*f*. = 9 n.s.				

**KM Phenotypes**			***KM Allotypes***		
1	73	73.00	*1*	0.1081	0.0120
-1	284	284.00	*3*	0.8919	0.0120
					
Sample size	357				

5* = 5,10,11,13,14 allotypes

n.s. not significant

*S.E*. Standard Error

Among the six *GM *haplotypes found in Galicia, the most highly represented was GM* 3 23 5* (0.498), followed by GM*1,17 23' 21,28 (0.199) and GM*3 23' 5* (0.181). The haplotypes GM*1,17 23' 5* and GM*1,17 23' 10,11,13,15,16, assumed to be sub-Saharan and north-Asiatic respectively, were also found.

Taken together, the European GM*3 5 haplotype (which includes GM*3 23' 5* and GM*3 23 5*) reached a value of 0.679 in Galicia. The GM*3 23 5* haplotype represents 73% of the global value for both haplotypes. Similar high frequencies of the GM*3 23 5* haplotype are found in Valencia (0.482) [21]. In contrast, Basques from Guipúzcoa [18] and Pasiegos (MPAS) [20] register the lowest frequencies of the GM*3 23 5* (0.383 and 0.341, respectively) of all Spanish samples studied to date. In other Mediterranean populations the GM*3 23 5* haplotype is also especially common [22-24].

The GM*1,17 23' 5* haplotype shows a peak in Galicia (0.045), although values of around 0.04 have also been found in the Aran valley in the Pyrenees [25] and the large islands of Sicily, Corsica and Sardinia [24]. In Spain, the average figure for this African haplotype is 0.024, whereas in other non-Mediterranean European populations that value is nearly eight times lower (0.003). Although some researchers have associated African traces in Iberia to Islamic invasions [12,13], the presence of GM*1,17 23' 5* haplotype in the Galician population may in fact be due to more ancient processes as well as more recent ones through the introduction of genes from black populations from America. Other evidence of African genetic influences in western Iberians has been provided by analysis of variation of Y chromosome [26] and mtDNA [27]. In the latter paper, the authors hypothesise that haplogroup L was introduced in Portugal through the recent Black African slave trade, while U6 lineages were associated with the Muslim rule of Iberia.

Within Iberia, the GM*1,17 23' 21,28 haplotype presented extreme values in Galicia (0.199) as well as in Basques (0.242–0.350). In the Mediterranean basin, frequencies of that haplotype were, in general, lower (0.107–0.176) than in Iberia. Gene diversity (*h*) estimates among Iberians varied within the range 0.462–0.569. In this study, the value of *h *for the sample from Galicia was 0.491 and so comparable to that observed in their Portuguese neighbour (0.462). In Basques from Spain, the heterogeneity values for *GM *haplotypes ranged from 0.510 to 0.551. All those gene diversities are referred to data on GM*3 5 haplotype without distinguishing in its two components as though the G2M (23) allotype was used.

**Figure 2:**
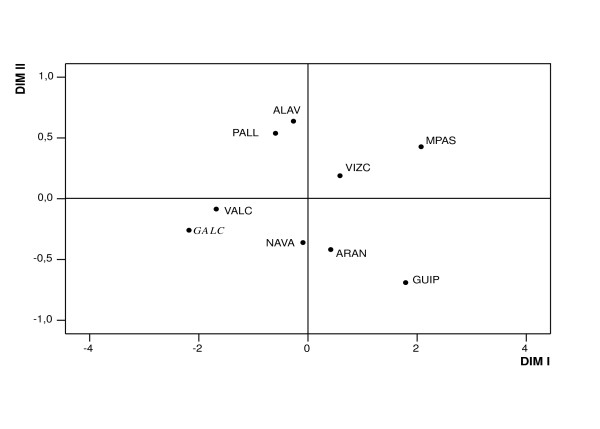
Multidimensional Scaling Analysis (MDS) based on the variation of seven *GM *haplotypes and using Reynolds's *F*_*ST *_distances among nine Spanish populations. All the samples were typed for GM2 (23) allotype, what it has allowed considering GM*3 23' 5* and GM*3 23 5* haplotypes separately. The study population is shown in italic.

MDS (*Stress value *= 0.0069) performed on Reynold's *Fst *genetic distances shows the genetic affinities among Spanish populations (Figure 2). In the genetic map it can be observed that Dimension I determines the presence of three groups: (VALC+GALC), (GUIP+MPAS) and other population samples (three of them are Basques) which are spread over the central zone of the first axis. The absence of a significant correlation between the geographic and genetic distances (*r *= 0.30, *P *= 0.105) for that set of populations shows that isolation by distance is not a direct factor explaining the observed Iberian genetic diversity. The four Basque subpopulations appeared in different quadrants, as was the case for the two geographically close Catalan Pyrenean subpopulations. Interestingly, Galicia was genetically close to Valencia, even though they are 750 km apart (see Figure 1). For the 32 pairwise comparisons between populations, the highest genetic distances observed were between GALC and GUIP (0.029) and between GALC and MPAS (0.032), with significant *F*_*ST *_(*P *values < 0.05), while the distance between GALC and VIZC was close to the cut-off for statistical significance (*P *= 0.07).

**Figure 3:**
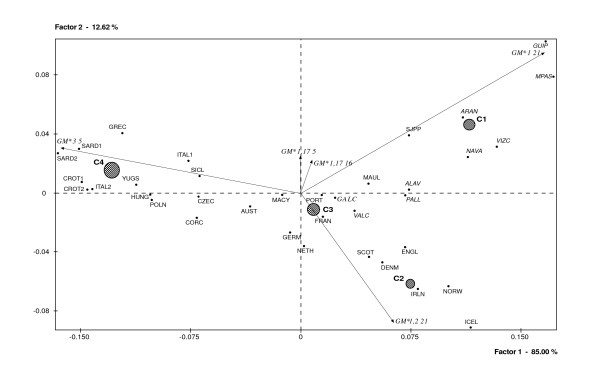
Principal Component and Hierarchical Cluster analyses based on the geographical variations of six *GM *haplotypes among 36 European populations. All Iberian samples are integrated in clusters C1 and C3. Black circles are proportional to the number of populations of each cluster. *GM *haplotypes have been written in a short hand designation.

The principal component analysis (PCA) shown in Figure 3 explores population relationships among a set of 36 European populations, including the Iberian group from this study (see Table 2). The Factor I and II account for 97.6% of the total genetic variance. The GM*1 21 and GM*3 5 haplotypes contribute significantly to Axis 1, whereas GM* 1,2 21 and to a much lower extent, the GM*1,17 5 and GM* 1,17 16 haplotypes do so for Axis 2. The hierarchical cluster analysis based on the first two factors displays the European populations organized into four clusters that produce an interesting Y-shaped configuration within the multivariate space. Cluster C1 displays an eccentric position and includes to the Basques from Spain, another French Basque sample from France (SJPP), the Pasiegos (MPAS) and the two Catalan samples. The high frequencies of GM*1 21, GM*1,17 16 and GM*1,17 5 haplotypes and the low levels of the GM*3 5 (GM*3 23' 5* + GM*3 23 5* haplotypes) European marker characterize the clustering of these populations in relation to the rest. Northern Europeans (Scandinavia, British Isles and Iceland) appear grouped in cluster C2, which is characterised by high frequencies for GM*1,2 21 haplotype and low frequencies for GM*3 5 and GM*1,17 5 haplotypes. The large cluster C4 is an intermingling of populations from Italy, the large Mediterranean islands and central and southeastern Europe, which would represent the Byzantine world and a certain Slavic component. This grouping is conditioned by the high incidence of GM*3 5 and low frequencies of GM*1,2 21 and GM*1 21 haplotypes. Finally, cluster C3, characterised by intermediate *GM *haplotype frequencies, is the nearest to the centroid and groups Western Europeans from France (including most French Basques), Germany, Austria and the Netherlands with the Iberian populations from Portugal, Galicia and Valencia. It is important to note that in this cluster there is a high genetic proximity between Galicia and Portugal, its geographic neighbour, which for centuries shared a common history, but also with France, geographically distant from Galicia and with a different history.

**Table 2:** The group of Iberian and other European populations selected for genetic structure analysis based on *GM *haplotype geographical variations.

	**Populations**	**Acronym**	**References**
	***Iberian Populations***		
1	**Galicia (Spain)**	***GALC***	***Present Study***
2	Montes de Pas (Spain)	MPAS	20*
3	Vizcaya Basques (Spain)	VIZC	18
4	Álava Basques (Spain)	ALAV	18
5	Guipúzcoa Basques (Spain)	GUIP	18
6	Navarra Basques (Spain)	NAVA	19
7	Pyrenean Aran Valley (Spain)	ARAN	42
8	Pyrenean Pallars Sobirá (Spain)	PALL	25
9	Valence (Spain)	VALC	43
10	Portugal	PORT	21
			
	***Other European Populations***		
11	Macaye Basques (France)	MACY	44
12	St. Jean P.P. Basques (France)	SJPP	44
13	Mauleon Basques (France)	MAUL	44
14	France	FRAN	45
15	England	ENGL	46
16	Scotland	SCOT	21
17	Ireland	IRLN	46
18	Germany	GERM	21
19	Netherlands	NETH	47
20	Austria	AUST	47
21	Hungary	HUNG	21
22	Poland	POLN	21
23	Czechoslovakia	CZEC	21
24	Denmark	DENM	46
25	Norway	NORW	21
26	Iceland	ICEL	21
27	Italy	ITAL1	21, 48
28	Pavia (Italy)	ITAL2	23
29	Sicily (Italy)	SICL	49, 21
30	Sardinia (Italy)	SARD1	50, 21
31	Sardinia (Italy)	SARD2	50
32	Corsica	CORS	45
33	Yugoslavia	YUGS	21
34	Croatia	CROT1	44
35	Pag Island (Croatia)	CROT2	22
36	Greece	GREC	21

*for details see Reference Section

Table 3 shows the analysis of molecular variance (AMOVA) for different levels of population groupings. The effect of considering GM*3 5 haplotype jointly or GM*3 23' 5* and GM*3 23 5* separately in their two components is notable for most of the *F*_*ST *_and *F*_*CT *_values. For the Spanish group, the *Fst *value is 0.0031 when GM*3 5 is considered jointly, whereas that parameter is 0.0121 (*P *< 0.001) when GM*3 23 5* and GM*3 23' 5* are distinguished. Thus, the results show that the GM2 (23) allotype increases considerably the resolution in the analysis of population genetic structure. The extent of genetic diversity among the 36 European populations showed a significant value of *F*_*ST *_= 0.0134 (*P *< 0.001) when GM*3 23' 5* and GM*3 23 5* were joined haplotypes. As many genetic studies have previously shown, European populations are quite homogeneous in relation to other continental groups. The small *F*_*ST *_value observed for the Iberian Peninsula accounted for 23% of the European one, but the area of the Iberian Peninsula is only 5% of that of Europe, and an even lower percentage would be found when the corresponding population sizes were compared.

**Table 3:** Fixation indices *F*_*ST *_and *F*_*CT *_of population genetic structure analysis based on *GM *immunoglobulin haplotypes.

**Groups (*K*)**	**Population Groups**		
		***F***_***ST***_	***F***_***CT***_
1	Spain (9)^a^	0.0031^ns ^(0.0121***)^b^	
1	Iberian Peninsula (10)	0.0041*	
1	Europe (36)	0.0134***	
			
2	Basques from Spain (4) + non-Basques from Spain (5)		0.0017^ns ^(0.0052)^b^
3	(GALC+VALC)^c ^+ (GUIP+MPAS)^c ^+ other Spanish populations (5)		0.0105** (0.0173**)^b^
2	Iberian Peninsula (10) + other European populations (26)		0.0081**

a = number of populations by group

b = values of *F*_*ST *_and *F*_*CT *_with GM* 3 23' 5* and GM* 3 23 5* haplotypes separately

c = acronyms given to populations shown in Table 2

n.s. not significant, *P < 0.05 **P < 0.01, ***P < 0.001

Other results concerning the degree of differentiation between groups (*F*_*CT*_) are also shown in Table 3. Spanish populations are divided into two and three groups, and again, results are provided for GM*3 23' 5*and GM*3 23 5* considered jointly or separately. The level of differentiation estimated between Basque and non-Basque Spanish populations were not significant. Nevertheless, when we form three groups corresponding to (GALC + VALC), (GUIP + MPAS) and the remaining Spanish populations, as suggested by the MDS plot, the level of differentiation was significant (*F*_*CT *_= 0.017, *P *= 0.02). Also, a significant level of differentiation was found among Iberian and other European populations. Dupanloup [28] noted that the identification of the correct number of groups depends critically on the degree of differentiation between groups and the absence of isolation by distance within groups, and should increase with the number of available loci. Thus, a certain degree of homogeneity in the characteristics of populations within groups (i.e. demographic sizes, historical processes, isolation, etc.) should be considered as another important facet in the analysis of human population genetic structure.

## Discussion

The current genetic structure of Iberian populations has presumably been affected by the complex orography of its territory, the different people and civilizations that settled there, its ancient and complex history, the diverse and persistent sociocultural patterns among its regions and the effect of the Iberian Peninsula representing a refugium area after the last glacial maximum (LGM, ~18000 BP). Natural selection has sometimes been addressed to explain geographic differentiations of *GM *haplotypes. However, in general, GM and disease association studies failed to demonstrate strong directional selective effects, or gave contradictory results. If some selection nevertheless played a role, the main effect would have been to enhance the magnitude of intercontinental differentiation without altering the global pattern of genetic relationships [[29], and references there in]. The variation of *GM *haplotype frequencies worldwide shows a strong geographic structure but also a significant isolation by distance. At a more local level, as addressed in the current study, *GM *haplotypes within Iberia do not appear to display a clear structure in relation to the geographic topology of the populations. Clearly, this result does not allow a simple interpretation, and may be due in part to the fact that the demographic size and degree of homogeneity of some of the populations analyzed is quite different, their number is somewhat limited and they are located in a small part of the peninsula (the majority are from the Cantabrian region in the north of Spain). Three of them, the population from Monte de Pas (MPAS) together with the Pyrenean populations from the valleys of Aran (ARAN) and Pallars Sobirá (PALL), represent mountain populations, which are characterised by a sustained mobility and small demographic sizes, with marked historical demographic changes that, in turn, can have shaped their own genetic structures [30]. Furthermore, mountain societies do not correspond adequately to their surrounding territories. This would be particularly the case of the *Pasiegos*, the native population of Montes de Pas (MPAS), a very small region situated in the Autonomous Community of Cantabria and neighboring the border with the province of Burgos, and therefore, very close to the Basque province of Vizcaya. This small enclave represents a population of only a few thousand people, descended from migrant herders who became established there around the 11th century and whose origin is unknown. The inbreeding levels registered among the inhabitants of Montes de Pas for the period 1850–1979 are high (F¯
 MathType@MTEF@5@5@+=feaafiart1ev1aaatCvAUfKttLearuWrP9MDH5MBPbIqV92AaeXatLxBI9gBaebbnrfifHhDYfgasaacH8akY=wiFfYdH8Gipec8Eeeu0xXdbba9frFj0=OqFfea0dXdd9vqai=hGuQ8kuc9pgc9s8qqaq=dirpe0xb9q8qiLsFr0=vr0=vr0dc8meaabaqaciaacaGaaeqabaqabeGadaaakeaacuWGgbGrgaqeaaaa@2DD9@ = 0.0061) and the incidence of first cousin consanguineous mating has been secularly high as well [31]. That condition of human isolation ascribed to this Cantabrian population seems to be also supported by their peculiar genetic characteristics emerging from Y-chromosome and mtDNA variation [32]. Consequently, there may be various explanations for the close genetic affinity observed between Pasiegos and Basques from Guipúzcoa for the *GM *system [18]. In terms of the two Pyrenean populations, their demographic size is slightly larger than that of MPAS but they also represent mountain societies, with a certain degree of mobility and limited genetic stability.

Galicia displays an interesting genetic profile for *GM *haplotypes defined by the high presence of the European Mediterranean GM*3 23 5* haplotype, the low frequency of GM* 1,17 23' 21,28 and the relatively high incidence of the sub-Saharan marker GM*1,17 23' 5* When comparing Galician with other Iberian and European populations, some interesting results have been found: a) the differential genetic distances observed between Galician and Basque populations, particularly with Guipúzcoa Basques, b) the close genetic affinities between Galicia and Valencia, and c) the centered position of Galicia in the European genetic map and its close proximity to Portugal, a finding that is in agreement with their shared histories and geography.

One of the aims of the genetic studies undertaken in Galicia has been to address the relationship with Basque populations, which represent the other end of the Cantabrian axis. The geographic distance (~700 km) separating these two historical mountainous northern Iberian territories would have represented an important barrier to gene flow. Available data on classical allele frequencies show that there are large statistically significant differences between those two populations but that convergence is not well enough demonstrated at the DNA level [12, 13, 16 among others]. Certainly, the four Basque subpopulations from Spain represent a variable degree of differentiation with Galician and other Iberian populations, Guipúzcoa representing the Basque province with the most genetic distinctiveness, a finding that would agree with the Basque linguistic map [18].

The close genetic similarity between Galicia, an Atlantic population, and Valencia, a Mediterranean one, is also unexpected as they are geographically separated by a long distance and represent quite dissimilar cultures with different histories. One of the main routes of human dispersion from Europe towards Iberia from the late Palaeolithic has been the corridor of the western Pyrenees. The logical direction of those migratory movements would have involved three main axes: one of them westward, along the Cantabrian cornice; another towards the Castilian central plateau, in a southward and westward direction crossing all over the region of Castile-Leon until reaching Galicia and northern Portugal; and a third route of gene flow following the Ebro valley towards the Mediterranean coast of Spain. Those continuous population movements are likely to have produced interesting processes of admixture among rather distant Iberian populations located within the geographic area of influence of that corridor. Taking as a reference a critical border area between Spain and France (in the Basque province of Guipúzcoa), and thus located inside the main passageway of communication of the western Pyrenees, the geographic distance of Galicia and Valencia to that point would be quite similar. Nevertheless, it will be of interest to examine how Iberian genetic population diversities correlate with geographic distances to that main Pyrenean entrance point for gene flow, when further genetic data from different genomic regions were available, despite the continued presence of the Basque distortion. Such an analysis would obviously need to be accompanied by more extensive sampling to cover the main, critical geographic regions of Spain and Portugal.

When Galicia is compared with other European populations by principal component analysis, our study sample is seen to be integrated within the central cluster, even though its geographic position in the European territory is extreme. This finding suggests that the population of Galicia is the result of a relatively balanced mixture of European populations or of the ancestral populations that gave rise to them. This would support the importance of the migratory movements that have taken place in Europe over the course of recent human history and their effects on the European genetic landscape.

## Conclusion

*GM *and *KM *allotypes show a poor correlation between geographic and genetic distances in Spanish populations. The Basque subpopulations from Spain exhibit a variable degree of differentiation in relation to Galicia, the Basques from Guipúzcoa being the most genetically distant. The close genetic similarity observed between samples from Galicia and Valencia, despite the geographical distance between them and their cultural and historical distinctiveness, may be explained by their being located at similar distances from the western end of the Pyrenees, the main passageway for communication between Spain and the rest of Europe, through which most Iberian gene flow would have passed. Likewise, the relatively high incidence of the African marker GM* 1, 17 23' 5* in Galicia may be the result of ancient admixtures as well as the recent introduction of genes from mixed black populations from America.

The Galician population is genetically close to the population of Portugal, its geographic neighbor, but also to that of France, which is further away. The central position of Galicia in the European genetic landscape, despite its extreme geographical location in the Iberian Peninsula, would support the decisive role of historic migratory movements in Europe.

## Methods

### Historical demography of Galicia

The census performed in 1787 showed a population size of 1 345 803 (46 inhabitants per km^2^), in 1900 it was 1 980 525 and in 2000 the population reached 2 731 900 (93 inhabitants per km^2^). The proportion of the Spanish population accounted for by Galicia has been reduced by half over the last two centuries (12.9% in 1787, 10.6% in 1900 and 6.8% in 2000) [33].

Human migration from Galicia has represented a critical demographic event. A significant migration to South America, mainly to Argentina, Brasil and México, occurred during the period between 1860 and 1936; that period also saw migration to other parts of Iberian Peninsula, such as Portugal, Andalusia and Castile. By the early 1950s, just when the process of modernisation began in Spain, another migration wave coming from Galicia was attracted by the industrialization taking place in the Basque Country, Madrid, and Catalonia, and similar migration occurred toward other parts of Western Europe. That considerable and sustained outward migratory flow has had a strong impact on inbreeding patterns within the Galician autochthonous population.

### Samples and laboratory analysis

A total of 357 blood samples were collected from autochthonous (up to third generation), healthy, non-biologically related Galicians. Sampling procedures met the requirements of anonymity and confidentiality of the Ethics Committee of the University of Santiago de Compostela. Sampling was carried out in the universities of Santiago de Compostela, La Coruña, Lugo, Orense and Vigo, and efforts were made to ensure spatial homogeneity throughout the region of Galicia in terms of the origin of the families of sampled individuals. Serum samples were obtained from peripheral venous blood, and after addition of 0.2% sodium azide were stored at -30°C in the Laboratory of Physical Anthropology at the University of Santiago de Compostela until typing was performed. All the sera were tested for *G1M (1,2,3,17), G2M (23), G3M (5,6,10,11,13,14,15,16,21,24,28) *and *KM *(1) immunoglobulin allotypic markers by using a classical haemagglutination inhibition method [34] in the Centre d'Anthropologie, Toulouse, France. In this paper, the Guideline for Human Gene Nomenclature (International System for Human Gene Nomenclature, ISGN) [35] has been used. *GM *haplotypes are represented in italics, allotypes are separated by commas, and the subclasses are separated by spaces. The notation *23' *indicates the sample was tested for G2M (23) allotype and found negative.

### Analysis of genetic data

*GM *haplotype frequencies were calculated with the maximum likelihood method [36] and the Hardy-Weinberg equilibrium was evaluated by chi-square test. Given that typing of the *KM *system only involved the use of the anti-KM (1), only two phenotypes: KM(1) and KM(-1) were observed. Consequently, the frequency of the KM*3 allele was estimated using the square root of the relative frequency of individuals with a KM (-1) phenotype, the complement corresponding to the frequency of the KM*1 + KM*1,2 alleles. Standard errors were calculated using the square root of *p*_*i *_(1-*p*_*i*_*)/2N*, where *p*_*i *_is the frequency of allele *i *and N the total number of typed individuals.

For multivariate and other genetic structure analysis, the following *GM* haplotypes were used: GM*1,17 23' 21,28; GM*1,2,17 23' 21,28; GM*3 23' 5*; GM*3 23 5*; GM*1,17 23' 5*, and GM*1,17, 23' 10,11,13,15,16. The *GM *haplotypes are written here in a simplified form such that 5* = 5,10,11,13,14.

*GM *haplotype frequencies registered in Galicia were compared with other European samples obtained from the literature, among which the following nine belong to Spain: Galicia (GALC); Montes de Pas (MPAS), a herder population from Cantabria; autochthonous Basques from Vizcaya (VIZC), Alava (ALAV), Guipúzcoa (GUIP), and northern Navarre (NNAV); two Catalan samples from the Pyrenean valleys of Pallars Sobirà (PALL) and Aran (ARAN); and one sample from Valencia (VALC) in the east of Spain. All of these population samples were tested for the G2M (23) allotype, meaning that frequencies for the GM*3 23'5* and GM*3 23 5* haplotypes are available. Unfortunately, this is not the case for most European samples.

Reynold's *F*_*ST *_genetic distances [37] were assessed for the Spanish population group. To represent the corresponding genetic distance matrix we applied a non-metric multidimensional scaling (MDS) analysis, which represents a generalization and is a complementary approach to principal component analysis (PCA). Pairwise genetic distances were calculated for the populations with the GENDIST program from PHYLIP 3.57c [38] and MDS was performed using the SPSS statistical package (*v*.13.0). European population relationships based on the geographic variations of *GM *haplotypes were also examined by principal component and hierarchical cluster (HCA) analysis using the SPAD (Système Portable Pour L'Analyse de Données) statistical program [39]. In the genetic map *GM *haplotypes have been written in short designation such as: GM*1,17 23' 21,28 (GM*1 21);GM*1,2,17 23' 21,28 (GM*1,2 21); GM*3 23' 5*+GM*3 23 5* (GM*3 5); GM*1,17 23' 5* (GM*1,17 5) and GM*1,17,23' 10,11,13,15,16 (GM*1,17 16).

The ARLEQUIN (*v*.3.01) program [40] was used to estimate gene diversity (*h*) by population, the genetic structure of Iberian and European populations from *F*-statistics indexes (*F*_*ST*_, *F*_*CT *_and *F*_*SC*_), and the correlation between geographic and genetic distance matrices (Mantel test) for the Spanish group.

Geographic distances were calculated from Universal Transverse Mercator (UTM) coordinates, taking the corresponding capital town as a reference when samples referred to a province. In the case of Galicia, as the sampling mimics the population of the whole region, the UTM coordinates of the weighted centre of the present Galician population was used. When both populations were located within the same UTM zone, their geographic distance was calculated as a common Euclidean distance *D_ij_*= [(x_i _- x_j_)^2 ^+ (y_i _- y_j_)^2^]^1/2^, where *D_ij_* is the distance between points *i *and *j*. Otherwise, UTM coordinates were transformed into geodesic coordinates to take into account the curvature of the earth. These calculations were performed with specific software [41].

## Authors' contributions

RC planned and took responsibility of writing the manuscript. TAV, RL and JF designed the sampling process and collected the blood samples in the Galician region. EG carried out the characterization of immunoglobulin markers in the Galician sample, JMD supervised the allotypic genetic data, prepared the DataBase on *GM *haplotype frequencies in European populations and made interesting suggestions while preparing the manuscript. AG-M and BA worked together in the genetic data analysis. All authors contributing to this study read and agreed the final manuscript.

## References

[B1] López-Cuevillas F, Otero R (1979). Prehistoria. Historia de Galiza.

[B2] Kruta V (1977). Los Celtas.

[B3] Risco V (1976). Manual de Historia de Galicia.

[B4] Varela TA, Lodeiro R, Fariña J (1997). Evolution of consanguinity in the Archbishopric of Santiago de Compostela (Spain) during 1900–1979. Hum Biol.

[B5] Gómez P (1990). Distribución espacio-temporal del coeficiente y frecuencias de consanguinidad y endogamia en el norte de la Península Ibérica. (Región Centro Cantábrica). Bol Cienc Mat IDEA.

[B6] Calderón R, Peña JA, Morales B, Guevara JI (1993). Inbreeding patterns in the Basque Country (Alava Province, 1831–1980). Hum Biol.

[B7] Aresti U (2006). Estructura y evolución de la consanguinidad en Guipúzcoa, 1862–1995. Efectos de la migración sobre el parentesco genético. PhD thesis.

[B8] Calderón R (1989). Consanguinity in the Archbishopric of Toledo, Spain, 1900–79. I. Types of consanguineous mating in relation to premarital migration and its effects on inbreeding levels. J Biosoc Sci.

[B9] Morales B (2002). Estructura de la consanguinidad en la Diocesis de Siguenza-Guadalajara. Variación histórica, microgeografía y genealogía. PhD thesis.

[B10] Blanco-Villegas MJ, Boattini A, Otero HR, Pettener D (2004). Inbreeding patterns in La Cabrera, Spain: dispensations, multiple consanguinity analysis, and isonymy. Hum Biol.

[B11] Gusmao L, Sanchez-Diz P, Alves C, Lareu MV, Carracedo A, Amorim A (2000). Genetic diversity of nine STRs in two northwest Iberian populations: Galicia and northern Portugal. I. J Legal Med.

[B12] Brion M, Quintans B, Zarrabeitia M, Gonzalez-Neira A, Salas A, Lareu V, Tyler-Smith C, Carracedo A (2004). Micro-geographical differentiation in Northern Iberia revealed by Y-chromosomal DNA analisis. Gene.

[B13] Flores C, Maca-Meyer N, González AM, Oefner PJ, Shen P, Pérez JA, Rojas A, Larruga JM, Underhill PA (2004). Reduced genetic structure of the Iberian Peninsula by Y-Chromosome analysis: implications for population demography. Eur J Hum Genet.

[B14] Roewer L, Croucher PJP, Willuweit S, Lu TT, Kayser M, Lessig R, Knijff P, Jobling MA, Tyler-Smith C, Krawczak M (2005). Signature of recent historical events in the European Y-chromosomal STR haplotype distribution. Hum Genet.

[B15] Antunez de Mayolo G, Antunez de Mayolo A, Antunez de Mayolo P, Papiha SS, Hammer M, Yunis JJ, Yunis EJ, Damodaran C, Martínez de Pancorbo M, Caeiro JL, Puzyrev VP, Herrera RJ (2002). Phylogenetics of worldwide human populations as determined by polymorphic *Alu *insertions. Electrophoresis.

[B16] Salas A, Comas D, Lerau MV, Bertranpetit J, Carracedo A (1998). mtDNA analysis of the Galician population: a genetic edge of European variation. Eur J Hum Genet.

[B17] Lefranc MP, Lefranc G, Shakib F (1990). Molecular genetics of immunoglobulin allotype expression. The Human IgG subclasses: molecular analysis of structure, function and regulation.

[B18] Calderón R, Vidales C, Peña JA, Pérez-Miranda A, Dugoujon JM (1998). Immunoglobulin allotypes (GM and KM) in Basques from Spain: Approach to the origin of the Basque population. Hum Biol.

[B19] Calderón R, Pérez-Miranda A, Peña JA, Vidales C, Aresti U, Dugoujon JM (2000). The genetic position of the autochthonous subpopulation of Northern Navarre (Spain) in relation to other Basque subpopulations. A study based on GM and KM immunoglobulin allotypes. Hum Biol.

[B20] Esteban E, Dugoujon JM, Guitard E, Sénégas MT, Manzano C, de la Rúa C, Valveny N, Moral P (1998). Genetic diversity in Northern Spain (Basque Country and Cantabria): GM and KM variation related to demographic histories. Eur J Hum Genet.

[B21] Steinberg AG, Cook CE (1981). The Distribution of the Human Immunoglobulin Allotypes.

[B22] Borot N, Dugoujon JM, Janicijevic B, Rudan P, Chaventre A (1991). GM and KM immunoglobulin allotypes in populations on the island of Pag (Croatia). Coll Antropol.

[B23] Lorini R, Orecchia G, Martinetti M, Dugoujon JM, Cuccia M (1992). Autoimmunity in vitiligo: relationship with HLA, GM and KM polymorphisms. Autoimmunity.

[B24] Cerruti N, Dugoujon JM, Guitard E, Rabino-Massa E (2004). GM and KM allotypes in Sicily. Immunogenetic.

[B25] Giraldo MP, Esteban E, Aluja MP, Nogués RM, Backés-Duró CH, Dugoujon JM, Moral P (2001). GM and KM alleles in two Spanish Pyrenean populations (Andorra and Pallars Sobira): a review of GM variation in the Western Mediterranean basin. Ann Hum Genet.

[B26] Pereira L, Joao-Prata M, Brion M, Jobling MA, Carracedo A, Amorím A (2000). Clinal variation of YAP^+ ^Y-chromosome frequencies in Western Iberia. Hum Biol.

[B27] Pereira L, Prata MJ, Amorim A (2000). Diversity of mtDNA lineages in Portugal: not a genetic edge of European variation. Ann Hum Genet.

[B28] Dupanloup I, Schneider S, Excoffier L (2002). A simulated annealing approach to define the genetic structure of populations. Mol Ecol.

[B29] Dugoujon JM, Hazout S, Loirat F, Mourrieras B, Crouau-Roy B, Sanchez-Mazas A (2004). GM haplotype diversity of 82 populations over the world. Am J Phys Anthropol.

[B30] Calderón R, Aresti U, Ambrosio B, Rosa JM (2005). Geographic, demographic and inbreeding patterns in a Basque mountainous region of Guipúzcoa. Int J of Anthrop.

[B31] Armiño-Macho MA, Gómez P (1987). Niveles de endogamia y consanguinidad en la población de los Montes de Pas (Cantabria, España). En Proceedings of V Congreso Español de Antropología Biológica: September 1987.

[B32] Maca-Meyer N, Sánchez-Velasco P, Flores C, Larruga JM, González AM, Oterinao A, Leyva-Cobián F (2003). Y chromosome and mithocondrial DNA characterization of Pasiegos, a human isolate from Cantabria (Spain). Ann Hum Genet.

[B33] Instituto Nacional de Estadística. http://www.ine.es/.

[B34] Field LL, Dugoujon JM (1989). Immunoglobulin allotyping (GM, KM) of GAW5 families. Genet Epidemiol.

[B35] Shows TB, MacAlpine P, Boucheix C (1987). Guidelines for human gene nomenclature. An international system for human gene nomenclature (ISGN). Cytogenet Cell Genet.

[B36] Edwards AWF (1984). Likelihood.

[B37] Reynolds J, Weir BS, Cockerham CC (1983). Estimation of the coancestry coefficient: basis for a short-term genetic distance. Genetics.

[B38] Felsenstein J (1995). PHYLIP (Phylogeny Inference Package) version 3.5c. Distributed by the author.

[B39] Lebart L, Morineau A, Warwick KM (1984). Multivariate descriptive statistical analysis: correspondence analysis and related techniques for large matrices. Series in Probability and Mathematical Statistics.

[B40] Excoffier L, Laval G, Scheneider S (2005). An integrated software package for population genetic data analysis. Evolutionary Bioinformatics.

[B41] Centro Nacional de Información Geográfica. http://www.cnig.es.

[B42] Giraldo MP, Vallet M, Guitard E, Sénégas MT, Sevin A, Nogués RM, Aluja MP, Dugoujon JM (1998). GM and KM immunoglobulin allotypes in a Spanish Pyrenean population: Val de Aran. Ann Hum Biol.

[B43] Schanfield MS, Baylerian R, Maiquez J, Carbonell F (1981). Immunoglobulin allotypes in European populations. IV. GM, AM and KM allotypic markers in Valencia, Spain. J Immunogent.

[B44] Dugoujon JM, Borot N, Chaventre A, Rudan P (1989). Gm and Km immunoglobulin allotypes in the Olib and Silba islands (Northern Dalmatia, Yugoslavia). Coll Antropol.

[B45] Blanc M, Ducos J, Ohayon E, Cambon-Thomsen A (1986). Les allotypes des systèmes GM et KM dans les provinces françaises.. Gènetique des Populations.

[B46] Roychoudhury AK, Nei M (1988). Human Polymorphic Genes and World Distribution.

[B47] Cavalli-Sforza LL, Menozzi P, Piazza A (1994). The History and Geography of Human Genes.

[B48] Larizza D, Martinetti Biachi M, Lorini R, Maghnie M, Dugoujon JM, Cuccia Belvedere M, Severi F (1989). Autoimmunity, HLA, GM and KM polymorphisms in Turner's syndrome. Autoimmunity.

[B49] Marziano E (1968). Distribuzione dei fattori serici gruppo specifici Gma-, Gmb-, Gmx-nella popolazione della Sicilia orientale. Med Leg J.

[B50] Piazza A, Van Loghem E, de Lange G, Curtoni ES, Ulizzi L, Terrenato L (1976). Immunoglobulin allotypes in Sardinia. Am J Hum Genet.

